# Growth kinetics of *Taylorella equigenitalis* and its survival in straw-based bedding

**DOI:** 10.1007/s11259-026-11278-1

**Published:** 2026-05-19

**Authors:** Martina Florianova, Miroslava Krzyzankova, Kristyna Korena, Helena Juricova

**Affiliations:** Department of Microbiology and Antimicrobial Resistance, Veterinary Research Institute, Hudcova 296/70, Brno, 621 00 Czech Republic

**Keywords:** *Taylorella equigenitalis*, Growth kinetics, Extra-host survival, Persistence

## Abstract

*Taylorella equigenitalis*, the causative agent of contagious equine metritis, is a fastidious bacterium with limited tolerance to extra-host conditions; however, quantitative data on its growth dynamics and survival outside the host remain scarce. This study evaluated the growth of *T. equigenitalis* under aerobic and microaerophilic conditions in liquid medium and its survival in wet and dry straw-based bedding under controlled laboratory conditions.

Growth was assessed at 20 °C and 37 °C using real-time optical density monitoring and viable cell counts. No proliferation was observed at 20 °C, whereas incubation at 37 °C resulted in measurable growth after 25–33 h. Viable cell counts were higher after incubation under aerobic conditions than under microaerophilic conditions, with a mean difference of 0.45 log_10_. Bacterial survival in straw-based bedding declined progressively over seven days, with significantly higher counts maintained under wet conditions compared to dry conditions. Viable cells remained detectable until the end of the experiment, particularly in wet bedding.

The results provide quantitative data on the growth characteristics of *T. equigenitalis* and underscore the importance of appropriate hygiene and biosecurity measures in equine breeding facilities.

## Introduction

*Taylorella equigenitalis* is the causative agent of contagious equine metritis (CEM), a venereal disease of equids with significant implications for animal health, breeding management and international trade (Schulman et al. 2013; WOAH 2022). It is a Gram-negative, microaerophilic, capnophilic bacterium of the family Alcaligenaceae, whose primary habitats are restricted to poorly oxygenated parts of the equine reproductive tract, such as the urethral fossa, distal urethra, prepuce, and the free part of the penis in stallions, as well as the clitoral fossa and clitoral sinuses in mares.

*T. equigenitalis* is a slow-growing, fastidious bacterium that requires cultivation on chocolate agar under strictly controlled microaerophilic conditions, typically with 5–10% CO_2_ at 37 °C for 3–7 days. In addition to demanding culture requirements, the bacterium also exhibits limited tolerance to environmental stressors (Hébert et al. 2012; WOAH 2022). Consequently, special care is necessary during sample collection, transport and processing. A progressive reduction in bacterial viability during transport has been documented, even when recommended transport media and conditions are applied (Duquesne et al. 2021).

Although the challenges associated with sample handling are well recognised, detailed experimental data on the survival and persistence of *T. equigenitalis* under unfavourable or extra-host environmental conditions remain scarce. In particular, information regarding its ability to survive or maintain viability outside the equine urogenital tract is limited. Such knowledge is, however, essential not only for improving diagnostic procedures but also for the development of effective biosecurity and zoo-hygiene management practices aimed at preventing the environmental dissemination and indirect transmission of the pathogen within equine breeding facilities (Grabatin et al. 2025; Schulman et al. 2013; Timoney 2011). Therefore, the aim of this study was to evaluate the viability of *T. equigenitalis* by assessing its growth potential under aerobic and microaerophilic conditions, as well as its survival in straw-based bedding, which represents a simple and standardizable abiotic matrix with a potential risk of contamination in the vicinity of infected animals.

## Materials and methods

### Bacterial strain

A *T. equigenitalis* TE31 isolate was isolated in 2021 during mandatory routine veterinary screening performed at the national stud farm in Kladruby nad Labem, Czech Republic (Hrala et al. 2025). For all laboratory experiments, the isolate was pre-cultivated on chocolate agar plates supplemented with nicotinamide adenine dinucleotide and hemin (LabMediaServis, Czech Republic) in a Brewer anaerobic jar for 48 h at 37 °C. A microaerophilic, CO₂-enriched atmosphere was generated using the candle jar method.

### Growth of *T. equigenitalis* under aerobic and microaerophilic conditions

The growth kinetics of the *T. equigenitalis* TE31 were evaluated in Bolton broth in an RTS-1 C Personal Bioreactor (Biosan, Latvia) under aerobic conditions. Bolton broth (Oxoid, UK) was prepared according to the manufacturer´s instructions. No selective antibiotic supplement was added. A fresh *T. equigenitalis* suspension was prepared in phosphate-buffered saline (PBS), adjusted to an optical density of OD_600_ = 0.5 and diluted 10,000 times. 1 mL of the dilution was used to inoculate 29-mL aliquots of Bolton broth. Following inoculation, one aliquot was incubated at 20 °C, while the second was incubated at 37 °C. The cultures were incubated in bioreactors with a spinning speed of 500 rpm and real-time optical density monitoring for a total duration of 50 h. The same setting was used for cell count determinations with aliquots incubated in parallel under aerobic and microaerophilic conditions. Viable counts of *T. equigenitalis* were determined at the beginning and at the end of the incubation period by plating tenfold serial dilutions in PBS onto chocolate agar plates. The growth experiments were conducted in three independent replicates. Bacterial growth was quantified as the fold change in cell counts over time, calculated as the ratio of the geometric mean CFU/mL at 50 h to the geometric mean CFU/mL at 0 h, and subsequently log_10_-transformed.

### Survival of *T. equigenitalis* in straw-based bedding

Laboratory bottles containing 5 g of straw-based bedding were sterilised twice by autoclaving at 121 °C for 15 min. A fresh *T. equigenitalis* suspension was prepared in PBS and adjusted to an optical density of OD_600_ = 0.5. Subsequently, 40 mL of the suspension was used for inoculation to simulate wet conditions, while 5 mL was used for inoculation to simulate dry conditions. The bottles were vigorously shaken for 1 min to ensure homogenous distribution of the inoculum within the bedding and incubated half-open at 20 °C. For each condition, eight bottles were prepared per replicate, with one bottle assigned to each sampling day (days 0–7). The experiment was conducted in three independent replicates. Under wet conditions, 1 mL of liquid was collected from the respective bottle at each time point, serially diluted tenfold in PBS, and spread onto chocolate agar plates for cell count determination. Under dry conditions, 20 mL of PBS was added to the respective bottle, followed by vigorous shaking for 1 min for *T. equigenitalis* extraction. The initial dilution step accounted for the volume of liquid added for extraction, after which standard tenfold serial dilutions were performed. The mean CFU/mL values were log-transformed, and the differences between wet and dry bedding were calculated for each day (0–7). The normality of these differences was verified using the Shapiro-Wilk test (shapiro.test; R project v4.5.2, stats package). Subsequently, the differences in the survival of *T. equigenitali*s in wet versus dry bedding were assessed by using a paired t-test (t.test; R project v4.5.2, stats package). The survival rate (SR, %) was calculated as the ratio of CFU/mL determined on a given day to the CFU/mL determined on day 0, multiplied by 100.

## Results

**Fig. 1:**
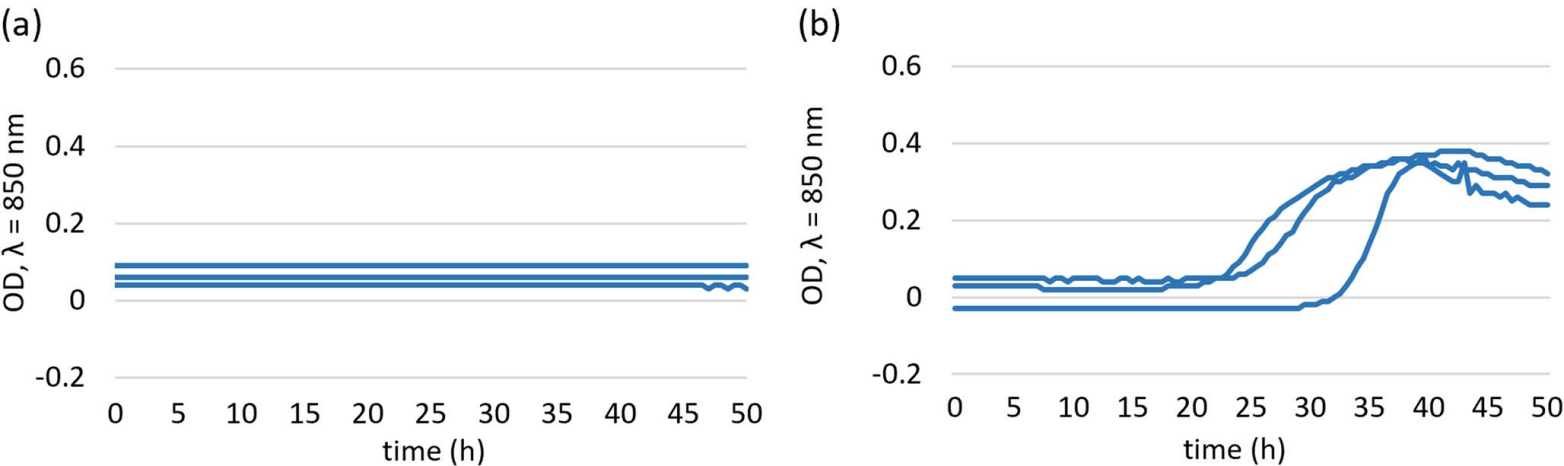
Growth curves of *T. equigenitalis* TE31 in Bolton broth under aerobic conditions at 20 °C (**a**) and 37 °C (**b**). In one replicate, the initial bacterial counts were slightly lower; however, the overall growth pattern was comparable across replicates

The growth kinetics of the *T. equigenitalis* TE31 isolate were evaluated in Bolton broth under aerobic conditions at two incubation temperatures. At 20 °C, the optical density remained close to baseline throughout the whole incubation period. Incubation at 37 °C resulted in a clear increase in optical density, with growth curves displaying a gradual exponential phase after approximately 25–33 h and a plateau reached toward the end of the incubation period (Fig. 1).

Viable cell counts were determined in a parallel experiment under aerobic and microaerophilic conditions. At 20 °C, bacterial counts decreased slightly throughout the incubation period, corresponding to a net change of − 0.49 log_10_ and − 0.38 log_10_ under aerobic and microaerophilic conditions, respectively. At 37 °C, a substantial increase was observed under both conditions. Bacterial counts increased from 7.25 × 10^3^ to 1.46 × 10^8^ under aerobic conditions and to 5.08 × 10^7^ under microaerophilic conditions, corresponding to a net increase of 4.3 log_10_ and 3.85 log_10_, respectively (Table 1).

**Table 1:** Mean viable cell counts and growth potential of *T. equigenitalis* TE31 after aerobic and microaerophilic cultivation at 20 °C and 37 °C in Bolton broth. Growth was higher under aerobic conditions than under microaerophilic conditions, with a mean difference of approximately 0.45 log_10_. Due to the loss of one replicate (aerobic cultivation, 37 °C, 50 h) caused by contamination, no formal statistical comparison between conditions was performed

Incubation temperature	Condition	Time (h)	G Mean CFU/mL ± SD	Growth log_10_ fold increase
20 °C	aerobic	0	7.25 × 10^3^ ± 1.12	-0.49
		50	2.36 × 10^3^ ± 1.19	
	microaerophilic	0	7.25 × 10^3^ ± 1.12	-0.38
		50	3.03 × 10^3^ ± 1.04	
37 °C	aerobic	0	7.25 × 10^3^ ± 1.12	4.3
		50	1.46 × 10^8^ ± 1.05	
	microaerophilic	0	7.25 × 10^3^ ± 1.12	3.85
		50	5.08 × 10^7^ ± 1.18	

**Fig. 2:**
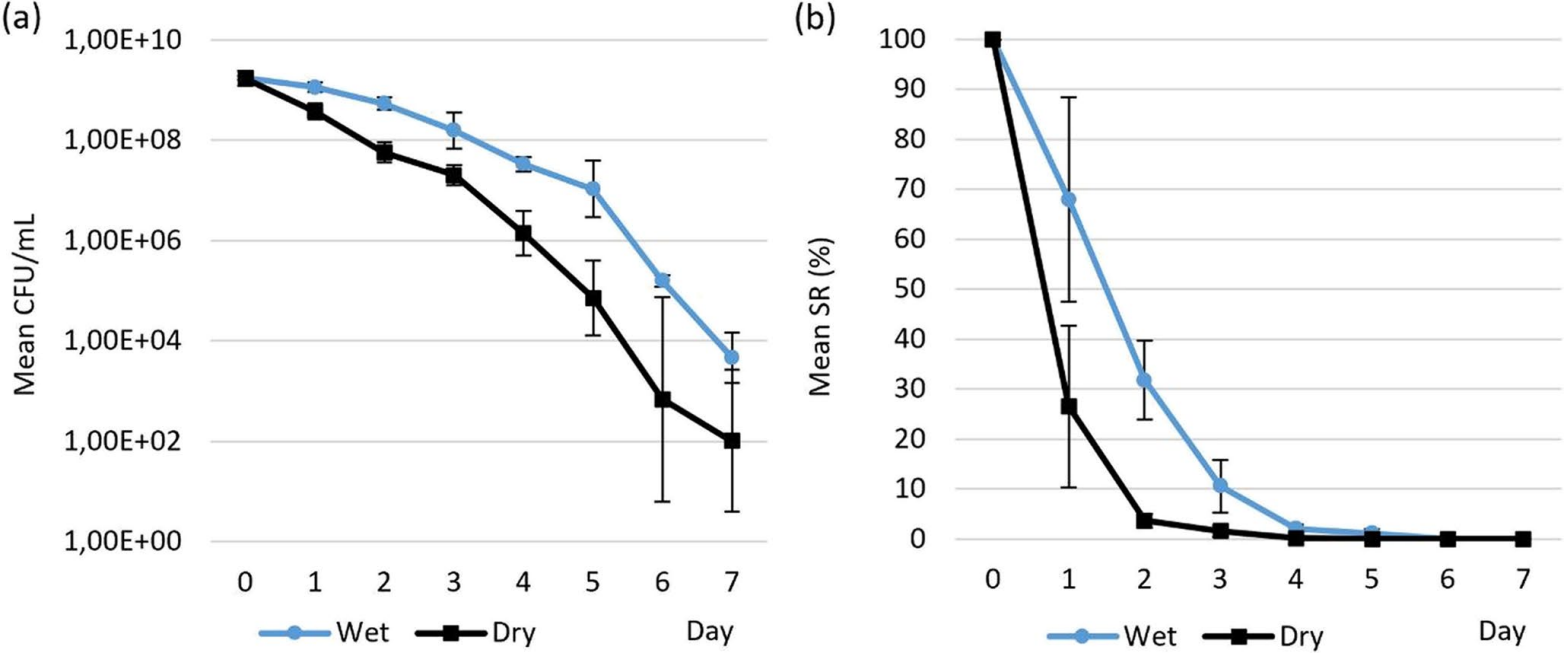
Survival of *T. equigenitalis* TE31 in wet and dry straw-based bedding stored aerobically at 20 °C. The data are presented as the geometric mean of cell counts (CFU/mL) ± SD (**a**) and as mean survival rate (%) ± SD relative to the initial bacterial load on day 0 (**b**) of three independent experiments. The mean difference between conditions across all time points was statistically significant (*P* < 0.01; paired t-test)

The survival of the *T. equigenitalis* TE31 isolate in straw-based bedding under wet and dry conditions was monitored over a period of seven days. Under both experimental conditions, a progressive decline in bacterial viability was observed over time, from 1.74 × 10^9^ on day 0 to 4.69 × 10^3^ and 1.02 × 10^2^ at the end of the experiment under wet and dry conditions, respectively (Fig. 2a). CFU counts were consistently higher in wet bedding than in dry bedding. The mean difference between conditions across all time points exceeded one order of magnitude (1.24 log_10_) and was statistically significant (*P* < 0.01; paired t-test). The mean SR decreased to 67.9% after 24 h and to 31.8% after 48 h in wet bedding. Viability continued to decline gradually over the following days, reaching 10.6% on day 3 and 2.1% on day 4. Low but detectable survival was still observed on day 7. In contrast, *T. equigenitalis* TE31 survival decreased more rapidly in dry bedding. The mean SR dropped to 26.6% after 24 h and to 3.7% after 48 h. By day 3, viability was reduced to 1.6%, and by day 4, the mean SR had fallen below 0.1%. On day 7, viable bacteria were detected in only one of the three replicates (Fig. 2b).

## Discussion

This study evaluated the growth of *Taylorella equigenitalis* under aerobic and microaerophilic conditions and its survival in straw-based bedding under controlled laboratory conditions. Incubation at 20 °C did not support active proliferation of *T. equigenitalis*, whereas incubation at 37 °C resulted in substantial growth in Bolton broth under aerobic conditions, characterised by a gradual exponential phase and a subsequent plateau. Notably, viable cell counts were higher after incubation under aerobic conditions compared to microaerophilic conditions.

Although *T. equigenitalis* is typically described as a fastidious bacterium requiring enriched media and microaerophilic, CO₂-enriched conditions, the present results confirm that growth in liquid medium can occur under strictly aerobic conditions. Similar observations have been reported previously (Fernie 1978). Bolton broth is commonly used in food microbiology for the enrichment of *Campylobacter* species, whose growth requirements are similar to those of *T. equigenitalis*. In the present study, bacterial growth was achieved without the addition of horse serum or other supplements, suggesting that this medium can support the proliferation of *T. equigenitalis* under less restrictive conditions than traditionally assumed.

The environmental resistance of *T. equigenitalis* is generally considered to be limited; however, quantitative data on its survival outside the host remain scarce. Previous studies have demonstrated short-term survival on fomites contaminated with reproductive secretions, such as semen collection equipment, supporting the possibility of indirect transmission in equine breeding settings (Schulman et al. 2013; Timoney 2011). In addition, the ability of *T. equigenitalis* and related *Taylorella* species to persist within environmental amoebae has been described, suggesting that protozoa could serve as a transient reservoir outside the equid host (Allombert et al. 2014).

Our study provides quantitative data on the survival potential of *T. equigenitalis* in an abiotic substrate resembling common stable bedding under typical environmental temperatures. Limited but detectable survival was observed in both wet and dry straw-based bedding, with viable bacteria recovered up to seven days after inoculation. Straw-based bedding is not considered a primary vehicle of CEM transmission; however, urine-contaminated bedding may represent a potential source of infection, given that *T. equigenitalis* colonizes the distal parts of the urogenital tract, potentially leading to contamination of the surrounding environment, including bedding.

Although the experimental conditions do not fully reflect the complexity of on-farm environments, these findings are consistent with the current understanding of *T. equigenitalis* ecology and underscore the importance of strict hygiene and biosecurity measures in equine facilities. The survival of low levels of viable bacteria suggests that naive animals could remain at risk of indirect exposure if thorough cleaning and disinfection are not implemented. Indirect transmission via fomites, breeding equipment or contaminated bedding has been implicated in outbreaks of contagious equine metritis (Deleure et al. 2019; Harpke et al. 2026; Solbach et al. 2025). These findings highlight the importance of proper hygiene practices during breeding, semen collection and stable management to prevent the spread of contagious equine metritis.

## Data Availability

No datasets requiring storage in public databases were generated. The data are available from the corresponding author on request.
